# Replicon: a software to accurately predict DNA replication timing in metazoan cells

**DOI:** 10.3389/fgene.2014.00378

**Published:** 2014-11-03

**Authors:** Yevgeniy Gindin, Paul S. Meltzer, Sven Bilke

**Affiliations:** ^1^Genetics Branch, Center for Cancer Research, National Institutes of HealthBethesda, MD, USA; ^2^Graduate Program in Bioinformatics, Boston UniversityBoston, MA, USA

**Keywords:** DNA replication, cell cycle, mathematical modeling

## Abstract

Eukaryotic DNA replication follows a strict temporal program where genomic loci are replicated at precise times during the S phase of the cell cycle. Yet, the mechanism in control of the timing program in metazoan cells is poorly understood. In a recent publication, the authors proposed an intuitive stochastic model of DNA replication and showed that it predicts replication timing with an accuracy approaching the level of experimental biological repeats. Here, we discuss an extended software implementation of the mechanistic model: Replicon. This package allows interested researchers to predict the global replication timing program in human cells from chromatin data.

## 1. Introduction

The strict temporal order of DNA replication in metazoan cells has been observed for over half a century (Taylor, 1960). Yet, relatively little is known about what governs this process (for recent review, see Masai et al., 2010). Studies of fission yeast suggest that DNA replication is initiated in a time-stochastic manner, where the global temporal patterns of DNA replication timing arise from uncoordinated initiation events (Dai et al., 2005). While DNA replication timing has been modeled successfully in fission yeast (Hyrien and Goldar, 2010), no such model has existed for metazoan cells.

Among the roadblocks to modeling DNA replication timing in metazoan cells has been the lack of a comprehensive catalog of the locations and amplitudes of replication initiation (Martin et al., 2011; Besnard et al., 2012). Yet, even if such a catalog were to become available, the resulting model would require more than 100,000 parameters (Pope et al., 2013) (at least one per initiation site)—a process that would have to be repeated for new cell lineages and species.

Recently we have shown that DNA replication initiation sites are sufficiently localized by DNase hypersensitivity (HS) and that the amplitude of replication initiation at individual sites has negligible effect on the global DNA replication timing program (Gindin et al., 2014). The current work showcases a vastly expanded computational model of DNA replication timing in human cells—Replicon. To the best of our knowledge, Replicon is the only mechanistic model of replication timing in mammalian cells. Unlike any other model, it can predict, rather than reproduce, replication timing because our model has no free parameters (with the exception of one technical constant). We have shown earlier for human cells that based only on the knowledge of DNase hypersensitive sites, our model accurately predicts replication timing for arbitrary cell types (Gindin et al., 2014).

There are a number of mechanistic models for the simpler genomes of various yeast strains (See Hyrien and Goldar, 2010, for a recent review). They all rely on a detailed characterization of replicators, the sites of replication initiation. In addition to replicator locations, typically two more parameters are required per site, firing efficiency nd average firing time. Unfortunately, metazoan genomes may contain more than 100,000 initiation sites, and metazoan replicators remain relatively poorly characterized. To use these models for metazoan cells therefore requires not only to fit several 100,000 parameters in order to reproduce timing data, but this procedure would have to be repeated again for each cell type. The same is true for another interesting approach aiming at identifying a histone code of eplication timing in *Drosophila melanogaster* (Comoglio and Paro, 2014). Here, too, the authors were able to reproduce the timing behavior in a given cell type, but the authors found that model parameters had to be re-adjusted for every cell type.

Our earlier publication (Gindin et al., 2014) included a core-version of our timing simulator. Since then we have substantially extended this simulator and added a new software suite, RepliconWrench. The core simulator, Replicon now allows to observe events in single cells as well as the entire cell ensemble. We have added simulations for a number of new measurement devices, including “DNA combing” experiments (Herrick et al., 2002), nascent strand bubble-chip and bubble-seq experiments to measure local initiation rates (Martin et al., 2011; Valenzuela et al., 2012), time of replication initiation measurements as well as global replication timing experiments that include a flow sorter (Ryba et al., 2011). To facilitate large-scale analyses, we implemented RepliconWrench—a software accessory tool that generates DNA replication initiation probability landscapes, a required Replicon input (see Methods), by querying relational databases of genome annotations or processing genome interval files.

## 2. Materials and methods

**Figure 1:**
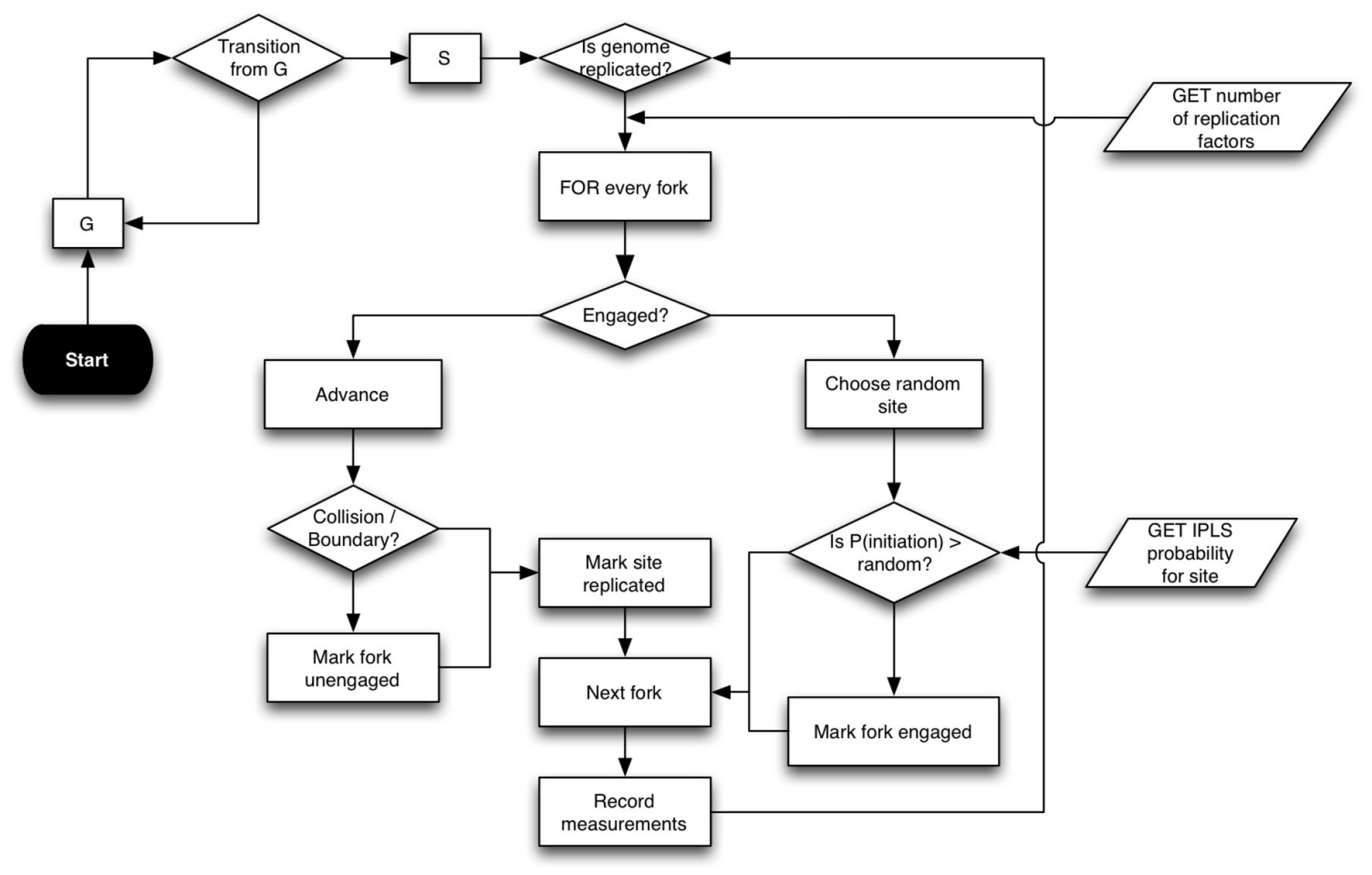
**Replicon algorithm flowchart**. Replicon predicts DNA replication timing by simulating cell-cycles in an asynchronous cell population. A simulated cell exists in either G (resting) or S (synthesis) state. While G to S transition occurs at random, the transition from S to G occurs upon completion of genome replication. Upon entering the S state, each cell queries the status of replication forks at its disposal. Forks that are engaged in replication are advanced by an interval (governed by IPLS resolution). Forks are disengaged if their advancement causes either a collision with another fork or if the fork reaches chromosome boundary. If the cell has at least two un-engaged forks at its disposal, then Replicon chooses a random unreplicated chromosome position and initiates replication with probability specified for that position in the IPLS. Replication then proceeds bidirectionally.

DNA replication is simulated on a cell population consisting of independent cells (Figure 1). A Replicon-simulated cell exists in two states: S, when it is actively replicating its genome; and G, when it is at rest. The simulation starts with all cells in G state. Cells transition from G to S following an interval drawn from a normal distribution. There are two required inputs to Replicon. The inputs to each cell are: (1) sites of potential DNA replication initiation events; and (2) a number of replication factors (see Table 1 for a complete list of command-line arguments).

**Table 1:** **Replicon command-line parameters**.

**Parameter name**	**Parameter action**
Flow-sorter	Comma-separated list of flow-sort boundaries
Indices	Measure S-phase and replicator engagement fraction
Initiate	Measure the global initiation rate as a function of S-phase fraction
Nascent	Simulate nascent strand measurements
Ncells	Number of cells (default: 1000)
Nfork	Number of replication forks (default: 50)
Nmeas	Number of measurements (default: 2000)
Ntherm	Number of thermalization sweeps (default: 80000)
Overwrite	Overwrite result file
Pprogress	Probability to move replication fork (ignored if 2nd column is present in landscape file) (default: 1)
Prelease	Probability to release replication fork (ignored if 3nd column is present in landscape file) (default: 0)
Singlemolecule	Simulate single molecule experiments
Stepsize	Sweeps per measurement (default: 20000)
Threads	Number of threads (default: 1)
Timing	Perform direct timing measurements

The first input specifies the number of replication factors that bind to initiation sites. These factors replicate DNA bi-directionally at a constant velocity of 50 bp/s. Replication factors are recycled when they collide with one another or when they reach a chromosome's physical boundary. The second input, Initiation Probability Landscape (IPLS), specifies the probability of replication initiation for every genome locus. At each step in the cell-cycle simulation, a replication factor chooses a random, unreplicated position in the genome and begins replication with a probability specified for that locus in the IPLS. In our earlier study (Gindin et al., 2014) we had shown that the choice of exact number of replication factors parameter is not critical and that using one fork per 80 MB yielded accurate replication timing predictions for human cells.

The IPLS file format is a simple two column file where the first column specifies a genome location and a second column specifies a probability of initiating replication at that location. Chromosome designation need not be specified in the IPLS file as Replicon simulates replication on one chromosome at-a-time. The choice of genome intervals in the IPLS files, which determines the resolution of the resulting replication time predictions, is user-driven. In our work, we found that a 500 bp IPLS resolution is well-suited for comparison with existing experimental data (see the accompanying Replicon web-site for a through description). IPLS files may be generated from “bedgraph” files either with a custom script or with a provided Replicon accessory tool—RepliconWrench. The RepliconWrench suite handles conversion of “bedgraph” or similarly formatted files into chromosome-specific IPLSs. Additionally, RepliconWrench could be used to query relational databases and generate IPLSs directly from genome annotations, such as DNase HS sites.

Replicon is written in C++ and supports multi-threading. In our simulations, Replicon was executed on a moderately sized SGE-managed computing cluster. A whole-genome simulation of replication timing for human cells required 15 min when running on 22, 4-core 2.93 GHz Linux nodes (one node per chromosome). RepliconWrench is written in Java and is compatible with Java 1.5 or higher.

## 3. Results

Replicon's accuracy can be ascertained by calculating a correlation coefficient between its prediction and experimentally observed data. For that purpose we use the mouse ENCODE data (ENCODE Project Consortium and others, 2011) to obtain empirical DNA replication timing data. By way of demonstrating Replicon, we predict a DNA replication timing profile for mouse stem cells using DNase DGF data as input (Sabo et al., 2004) and compare the prediction with experimental observations (Hiratani et al., 2008).

**Figure 2:**
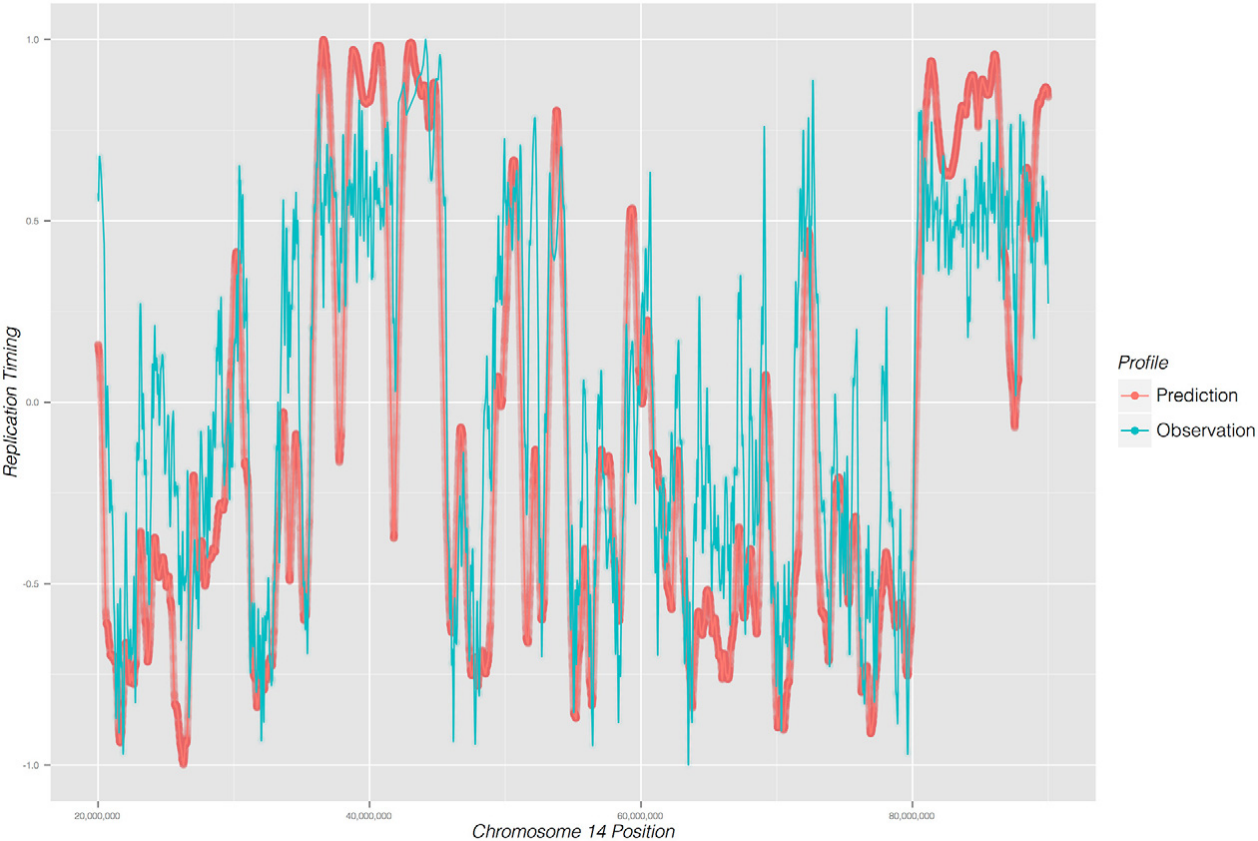
**DNA replication timing profile for a 65 mega-base region on chromosome 14 of mouse embryonic cells**. Replication time (early to late; y-axis) is plotted as a function of genome position (x-axis). Predicted profile (red) was generated from DNase I DGF data; experimentally observed timing profile is depicted in blue.

As illustrated in Figure 2, Replicon's predicted DNA replication timing profile closely reproduces experimentally observed DNA replication data. The correlation between Replicon's prediction and empirical data for mouse stem cells, averaged across 19 autosomal chromosomes, is 0.88 (root-mean-square deviation = 3.18). This is a remarkable level of prediction accuracy considering differing measurement approaches between simulation and experiment. Whereas Replicon calculates an expectation value for each genomic bin, empirical data are reported as a wavelet-smoothed ratios of early vs. late replication timing (Hiratani et al., 2008). Moreover, the quality of the Replicon's predictions are affected by experimental noise both from DNase DGF and replication timing experiments. These results signify the robustness of both the Replicon's algorithm and the DNA replication timing program.

**Figure 3:**
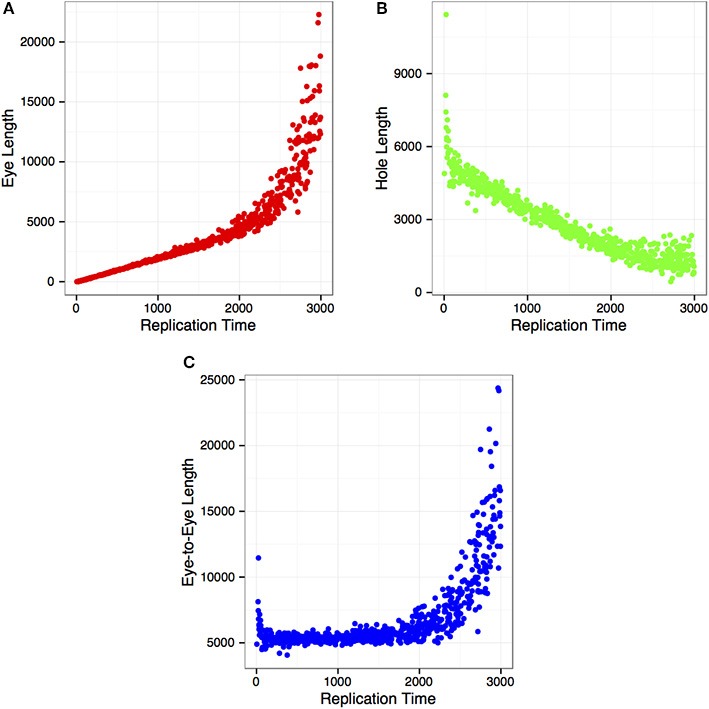
**DNA replication parameters as a function of time in S phase, illustrating; (A) replicon (eye) length; (B) length of unreplicated (hole) DNA; and (C) distance between replicon centers (eye-to-eye)**.

DNA combing technique has been used extensively to measure DNA replication kinetics on a single molecule scale. Herrick and colleagues, for instance, used this technique in an *in vitro* system to derive some of the quantitative parameters that govern DNA replication (Herrick et al., 2002). Here, we wanted to know if relationships between cell-cycle time and growth of newly synthesized DNA, observed and formulated by Herrick *et al*. in *Xenopus laevis*, could be recovered with the simulated DNA replication cycle performed by Replicon using *Mus musculus* DNase I hypersensitivity sites. To that end, we captured (Figure 3) the changing lengths of eyes (newly replicated), holes (yet-to-be replicated) DNA regions, and the distances between Replicon bubbles (eye-to-eyes) during the S phase. Our results match closely those observed by Herrick and colleagues. This is remarkable given that our simulation is based on mammalian cells, where DNA replication is initiated at well-defined loci, while, in *X. laevis*, DNA replication initiation sites are selected at random. This results further demonstrates that Replicon can be used to model single-cell observations of DNA replication such as those often performed using DNA combing.

## 4. Discussion

Replicon provides a way by which to accurately model DNA replication timing in metazoan cells. Here, we based our predictions on an IPLS derived from DNase I digestion data. Other IPLSs could just as easily be used to investigate, for instance, combinatorial effects of histone marks and DNA sequence motifs on DNA replication timing.

The association of replication timing with central cellular processes, such as differentiation or cytogenetic aberrations, has been revealed in recent years. But empirical replication timing data is not always available. In some instances it may not even be accessible if, for example, only formalin-fixed tissues is available. Given the close level of agreement between predicted and empirical DNA replication timing profiles, Replicon can be utilized to generate DNA replication timing predictions, provided appropriate genomic annotations are available.

## Availability and requirements

Project name: Replicon;

Project home page: https://github.com/RepliconBioinfo/;

Operating system: Platform independent;

Programming language: C++ and Java;

License: GNU;

Restrictions: None.

## Author contributions

Yevgeniy Gindin wrote the manuscript. Yevgeniy Gindin and Sven Bilke developed software and performed statistical analysis. Sven Bilke and Paul S. Meltzer conceived the study, supervised the work and edited the manuscript.

### Conflict of interest statement

The authors declare that the research was conducted in the absence of any commercial or financial relationships that could be construed as a potential conflict of interest.
